# A unique polysaccharide purified from *Hericium erinaceus* mycelium prevents oxidative stress induced by H_2_O_2_ in human gastric mucosa epithelium cell

**DOI:** 10.1371/journal.pone.0181546

**Published:** 2017-07-24

**Authors:** Mingxing Wang, Nakajima Kanako, Yanqiu Zhang, Xulang Xiao, Qipin Gao, Konishi Tetsuya

**Affiliations:** 1 Affiliated Hospital, Changchun University of Chinese Medicine, Changchun, Jilin, PR China; 2 Liaison R/D Center, Niigata University of Pharmacy and Applied Life Sciences, Niigata, Japan; 3 Research and Development Center, Changchun University of Chinese Medicine, Changchun, Jilin Province, PR China; University of PECS Medical School, HUNGARY

## Abstract

Hericium erinaceus (HE) has been used both as a traditional Chinese medicine and home remedy for treatment of gastric and duodenal ulcers and gastritis. EP-1, a purified polysaccharide isolated from HE mycelium, has recently been identified as the active component responsible for HE anti-gastritis activity. Because oxidative stress has been implicated as a pathogenic cause of gastritis and gastric ulcers, EP-1 antioxidant properties were systematically examined in vitro using the human gastric mucosal epithelial cell line, GES-1. Results showed that EP-1 possessed higher oxygen radical absorbance capacity (ORAC) and 2–3 times higher ability to scavenge 2,2-diphenyl-1-picrylhydrazyl (DPPH), superoxide and hydroxyl radicals than a hot water extract of commercially available HE fruiting body. A crude mycelial polysaccharide (CMPS) extract of HE, from which EP-1 was purified, showed slightly stronger radical scavenging activity and ORAC than EP-1, with the exception of DPPH-scavenging activity. Antioxidant activities of these extracts were further studied using hydrogen peroxide (H_2_O_2_)-abused GES-1 cells; EP-1 dose-dependently preserved cell viability of abused cells as assessed via MTT assay. Moreover, FACS analysis revealed that EP-1 prevented H_2_O_2_-induced apoptotic cell death by inhibiting activation of apoptotic cellular signals within mitochondria-dependent apoptotic pathways. CMPS also prevented H_2_O_2_-induced oxidative stress, but to a lesser degree than did EP-1, even though CMPS exhibited comparable or stronger in vitro antioxidant activity than did EP-1.

## Introduction

*Hericium erinaceus* (HE) is well known in Asia as a health-promoting food and important medicinal fungus. The anti-gastric/duodenal ulcer activity of the fruiting body has been well documented, as it has been widely used as a traditional Chinese medicine and home remedy for many years, particularly in Japan and China[1]. The HE fruiting body predominantly contains polysaccharides, several of which have been purified and their chemical structures elucidated[2]. Because previous research had demonstrated that a specific polysaccharide fraction isolated from the HE fruiting body exhibits anti-ulcer activity, this fraction should also be investigated for a potential role in the observed anti-gastritis activity of HE.

In a recent study, we isolated and chemically characterized a unique polysaccharide fraction from HE mycelium, designated EP-1, that exhibits anti-ulcer and anti-gastritis activity[3,4]. Gastritis is a clinically common gastrointestinal disease, with pathology characterized by oxidative stress and chronic inflammation[5]. Indeed,many studies have demonstrated that oxygen free radicals play an important role in the formation and development of gastritis and related diseases such as gastric cancer[6,7]. Although a few HE polysaccharide studies have demonstrated antioxidant activity[8], it has not yet been established if this activity is associated with HE anti-ulcer activity.

In the present study, the anti-oxidant property of both a crude mycelial polysaccharide (CMPS) extract of HE, as well as a purified polysaccharide fraction of EP-1, were systematically studied *in vitro* using GES-1 cells, a human musosal epithelial cell line. The results demonstrated that EP-1 exhibit significant antioxidant activities and protect GES-1 cells from H_2_O_2_-induced oxidative stress. This protective action is believed to function through enhancement of cellular antioxidant defenses mediated by glutathione (GSH) that result in modulation of apoptotic signal levels.

## Materials and methods

### Materials

Thiazolyl blue tetrazolium bromide (MTT; cat. no. M2128) was purchased from Sigma-Aldrich (St. Louis, MO, USA) and Annexin V conjugated Alexa Fluor 488 apoptosis detection kits (V-13245) were obtained from Molecular Probes, Inc. (Eugene, OR, USA). Primary antibodies against Bcl-2, Bax, Caspase 3, NRF2, Cytochrome c and β-actin as well as secondary antibodies were purchased from Santa Cruz Biotechnology, Inc. (Santa Cruz, CA, USA). COX IV was purchased from ProteinTech Group, Inc. (ProteinTech Group, Inc. Chicago, USA). The Bio-Rad protein assay kit II was supplied by Bio-Rad (Hercules, CA, USA) and the enhanced chemiluminescent western blot detection reagents (cat. no. RPN2106) were obtained from Amersham Pharmacia Biotech (Amersham, UK). As previously reported, ^1^ the polysaccharides (EP-1) used in this study were extracted from the HE mycelium, which complied with the National Drug Standards No. HI4023098 and purchased from Hangzhou Johncan Mushroom Bio-Technology Co., Ltd., Zhejiang, China. After freeze-dried, the polysaccharides were crushed into the powder and preserved in a desiccator. The other chemicals and reagents were analytical grade and obtained from local sources.

### Preparation and analysis of the samples

EP-1 was prepared from cultured mycelium of HE as described in our previous report[3]. Briefly, mycelium was extracted with hot water and then precipitated with 70–80% ethanol (CMPS). The CMPS fraction was successively subjected to hollow fiber ultrafiltration and ion–exchange chromatography to produce EP-1. Next, total carbohydrate was quantified using the phenol–sulfuric acid method with glucose (glu) as the standard and uronic acid was quantified using the m-hydroxydiphenyl method using glucuronic acid (glcA) as the standard. Protein content was determined by the Bradford method using bovine serum albumin (BSA) as the standard. Sugar components were analyzed by HPLC after converting the sugars into 1-phenyl-3-methyl-5-pyrazolone (PMP) derivatives. HPLC was performed using a Shimadzu GC-2010 instrument equipped with a C-18 column and controlled by a Uniport HP N-2000 data station. HEPS was methylated once using the Ciucanu method before performing HPLC.

### Mouse model for assessing the anti-gastritis effect

Forty mice (18–22 g, each group included 5 males and 5 females) were obtained from Jilin University, College of Pharmacy, with a certificate of conformity for the SCXK, (2012–0003). This study was carried out in strict accordance with the recommendations in the Guide for the Care and Use of Changchun University of Chinese Medicine. The protocol was approved by the Committee on the Ethics of Animal Experiments of Changchun University of Chinese Medicine(Permit Number: 20160307). All surgery was performed under sodium pentobarbital anesthesia, and all efforts were made to minimize suffering. The mice were randomly divided into the following 4 groups: normal control group, high dosage group (EP-1), low dosage group (EP-1), and positive control group. Each group was composed of 10 mice. The low dosage and high dosage EP-1 groups were administered with 1.2 and 2.5 g/kg respectively, directly into the stomach by oral gavage, once a day for 5 days. The positive control group was administered with 2.5 g/kg of CMPS. The control mice were administered with the same volume of distilled water. On the 5^th^ day of feeding, the mice were deprived of food but allowed free access to bottled tap water for 24 hours after the last dose and were then orally treated with 0.1 ml/20 g of anhydrous ethanol to induce gastritis. The animals were sacrificed 1 h later under anesthetized diethyl ether and their stomachs were quickly removed for further studies. The stomach of each experimental animal was opened along the large curvature and rinsed with distilled water to remove the gastric contents. The gastric ulcers appeared as elongated bands on the gastric mucosa with hemorrhagic lesions being parallel to the long axis of the stomach ^[^^6^^]^. The mucosa was assessed for damage under a dissecting microscope (1.8x), and a planimeter was used to measure the area of ulceration (hemorrhagic lesions). The length and the width of each lesion were measured, and the sum of the area of all the lesions for each stomach was expressed as the ulcer area (mm^2^). The ulcer area (UA) was measured, and the inhibition percentage (I%) was calculated by the following equation [4]:

$$I\%=\frac{UAcontrol-UAtreated}{UAcontrol}\times 100\%$$

### GES-1 cells culture

GES-1 cells (donated by Beijing University) are an adherent cell line and were cultured in Dulbecco’s modified Eagle’s medium (DMEM) supplemented with 10% calf serum (CS) at 37°C in a humidified atmosphere with 5% CO2. Flat-bottom plates were used for culture of GES-1 cells. Before the stimulations, the cells were synchronized using DMEM without calf serum for 12h.

### Radical scavenging activity determinations *in vitro*

DPPH(2,2-diphenyl-1-picrylhydrazyl) radical scavenging activity was determined using electron spin resonance (ESR) as described previously[9]. Briefly, the reaction solution consisted of an aliquot of an H_2_O extract of EP-1 or CMPS, 0.4 mM DPPH, and 100 mM Tris-HCl (pH 7.60) in 200 μl. The reaction was initiated by adding 100 μl of 0.4 mM DPPH and then the disappearance of DPPH radical signal was measured just 1 min after the addition of DPPH using a JEOL JES-TE 200 (X-band Microwave Unit) ESR spectrometer as described previously. The ESR measurement conditions were as follows: microwave power, 8 mV; microwave frequency, 9.43977 GHz; modulation amplitude, 32 m; time constant, 0.03 s; sweep time, 0.5 min; center fields, 341.0 mT.

Hydroxyl radical (·OH) scavenging activity was determined using a spin trapping ESR method with 5,5-dimethyl-1-pyrroline N-oxide (DMPO) as the spin trap reagent for ·OH generated by UV irradiation[10]. In the UV irradiation system, the reaction mixture, containing test samples dissolved in H2O, DMPO and H_2_O_2_, was placed into a hematocrit capillary tube and UV irradiated using a UVGL-58 handheld UV lamp (Funakoshi Co., Ltd., Japan) for 1 min. The DMPO-OH signal was recorded just 1 min after cessation of irradiation. ESR measurement conditions were as follows: microwave power, 8 mV; microwave frequency, 9.43977 GHz; modulation amplitude, 40 m; time constant, 0.03 s; sweep time, 0.5 min; center fields, 341.0 mT.

Superoxide anion radical scavenging activity was measured also by DMPO spin trapping ESR using a xanthine–xanthine oxidase system as a superoxide radical generator as previously reported[11]. To the reaction mixture, containing an aliquot of test sample in H2O, DMPO, 4 mM hypoxanthine, 5 mM DTPA in 300 μL total volume, 30 μL of an aqueous solution of xanthine oxidase (20 U/mL) was added to generate superoxide radicals. Next, the DMPO-OOH signal was determined using ESR just 1 min after the addition of xanthine oxidase. The ESR conditions were the same as described above except for use of the settings for field modulation width (0.2 mT) and modulation amplitude (100 m).

### Measurement of ORAC value

ORAC values were measured according to a method reported previously with slight modifications[12]. Stock solutions of fluorescein (4.19 × 10–5 mM), 2,2'-Azobis(2-amidinopropane (AAPH) as a peroxyl radical generator (153 mM) and Trolox as reference antioxidant were prepared in 75 mM potassium phosphate buffer (pH 7.4). To 25 μl of blank solution (75 mM potassium phosphate buffer) in a 96-well microplate, x μl of reference antioxidant (to achieve a final Trolox concentration of 5, 10, 25, 50 or 100 μM) or of experimental samples (0.25, 5.0, 1.0 or 2.0 mg/mL) and 150 μL of fluorescein were added successively. Following the addition of 25 μL of AAPH, the fluorescence intensity of the reaction solution was recorded at 37 oC for each 30-second interval for 90 min at the emission wavelength of 527 nm (excitation at 485 nm) using a fluorescence spectrophotometer (Labsystems Fluoroskan Ascent CF). ORAC values were calculated from the differences of areas under the fluorescence decay curves between the blank and either the sample or Trolox. The results were expressed as ORAC units, where 1 ORAC unit equals the net protection produced by 1 μM Trolox.

### MTT assay

Cell viability was determined using a standard assay using MTT, 3-(4,5-Dimethylthiazol-2-yl)-2,5-diphenyltetrazolium bromide, a common method for assessing cell viability[13]. Cells were plated at a density of 5 × 104 cells/well in 96-well plates and incubated with DMEM for 24 h. Next, the medium was replaced with medium containing EP-1 concentrations of either 0.1 or 0.5 mg/mL and incubated for 6 h. The cells were washed with PBS then treated with medium containing H_2_O_2_ (100 μM) for 12 h. Then 5 mg/mL MTT in phosphate buffered saline (PBS) was added to the cells in the plate for a final concentration of 1 mg/mL and incubated for 4 h at 37°C. The formazan formed by the reaction was dissolved in 150 μl dimethyl sulfoxide (DMSO) then the absorbance at 570 nm was measured using a microplate reader (Thermo Multiskan MK3, USA).

### Evaluation of apoptotic cells by Annexin V-FITC assay

GES-1 cells were plated into a 6-well plate at a density of 2 × 10^5^ cells/well and incubated overnight. Then HEPS extracts in DMEM were added to each well to final concentrations of 50 or 100 μg/mL and the cells were incubated for 6 h. After washing the cells with PBS, the cells were treated with 100 μM H_2_O_2_ for 12 h. The percentage of apoptotic cells was analyzed using a BD FACS Calibur flow cytometer (BD Biosciences, San Jose, CA, USA) after staining using an Annexin V-FITC Apoptosis Detection Kit (Biyuntian Biotech, Guanzhou, China) as described previously[14].

### Determination of GSH and MDA levels

According to the manufacturer’s instructions, the concentration of MDA and cellular GSH levels were determined using commercially available detection kits (Jiancheng Biological Engineering Academy, Nanjing, China).

### Western blot analysis

Cells were treated with two concentrations of test HEPS samples (0.1 or 0.5 mg/mL) in DMEM for 6 h, washed with PBS, then treated with DMEM containing H_2_O_2_ (100 μM) for 12 h. The cells were harvested, treated with lysis buffer (Thermo Fisher Scientific, MA, USA) and mitochondria were isolated using a Cell Mitochondria Isolation Kit (Beyotime Inst. Biotech, Peking, China). Protein concentrations in the extracts were quantified using the Bio-Rad Protein Assay reagent (Bio-Rad, Hercules, CA, USA) according to the manufacturer's instructions. An equal amount of protein (30 μg) for each sample was loaded onto SDS-PAGE gels (Bio-Rad), electrophoresed and transferred to 0.45 μm PVDF membranes (Millipore Corp., Billerica, USA) using a semi-dry transfer method. After blocking with 5% non-fat milk, the membranes were incubated overnight with corresponding primary antibodies at the following dilutions: Anti-Bcl-2, 1:500; anti-Bax, 1:1,000; anti-Caspase 3, 1:1,000; anti-NRF2, 1:1,000; anti-Cytochrome c, 1:1,000; COX IV, 1:1,000; and anti-β-actin, 1:2,000. Following incubation with appropriate secondary antibodies for 2 h at room temperature, antibody-bound protein bands were visualized using ECL detection reagents (Thermo Fisher Scientific, Rockford, IL, USA). The intensity of bands was quantified using an image analysis system. Results were expressed as the ratio of intensity of the target protein and that of the β-actin loading reference band in the same lane.

### Data analysis

SAS 6.0 was used for statistical analysis. Results are expressed as mean ± standard error of the mean (SEM). The analysis of variance (ANOVA) test was used for comparisons among the multiple groups. The mean values between two groups were compared using Student’s t-test. P<0.05 indicated statistical significance.

## Results

### Isolation and determination of EP-1

The mycelium of Hericium erinaceus was extracted with hot water and the extract was successively subjected to ethanol precipitation, hollow fiber ultra filtration and ion–exchange chromatography to obtain the purified anti-gastritis polysaccharide, designated EP-1, as described previously [1]. The carbohydrate content of EP-1 is 95.7% and the molecular weight is approximately 3 KDa, as determined by HPLC. The results of the methylation analyses of EP-1 obtained using GC-MS revealed that the glucosyl residues are mainly composed of (1→3)-linked Glup units with approximately 10% each of (1→)-Manp units and (1→3,4)-Glup units and 1.5% of (1→3,4)-Galp units.

### Anti-gastritis activity of EP-1 evaluated by an ethanol-induced gastritis model in mice

Gastric lesion measurements of ethanol-induced rats showed that EP-1 significantly reduced ulcer formation at the doses of 1.5 and 2.5 mg/kg in a dose-dependent manner (Table 1). Therefore, EP-1 was effective for the protection of mucosal injury induced by ethanol.

**Table 1:** Ulcer area measurements and the dose dependent ulcer inhibition effects of the EP-1.

groups	n	Ulcer area(mm^2^)	Ulcer inhibition percentage (%)
Normal control group	10	18.04 ± 0.91	
CMPS group	10	16.11 ± 0.81^*^	10.70
EP-1(2.5 g/kg)	10	14.16 ± 0.54^**^	21.51
EP-1(1.2 g/kg)	10	15.87 ± 0.87^*^	12.03

All the data were expressed as mean±SD of the mean of ten mice performed in triplicate.

* denoted P< 0.05, LD group and positive control group respectively VS normal control group

** denoted P< 0.01, HD group of EP VS normal control group

### Radical scavenging activity and ORAC of EP-1 determined *in vitro*

Radical scavenging activities of EP-1 and CMPS towards DPPH, superoxide radicals and hydroxyl radicals, as determined by the ESR method, are summarized in Table 2 together with ORAC values.

**Table 2:** DPPH, superoxide radicals and hydroxyl radicals scavenging activity of CMPS and EP-1.

Sample	ORAC value (μmol-Trolox/100g)	DPPH radical scavenging activity(μmol-Trolox/100g)	OH radical scavenging activity (μmol-Trolox/100g)	Superoxide anion radical scavenging activity (μmol-Trolox/100g)
CMPS(0.1mM)	14405±2946.1	1070.6±148.22	17737±1403.6	1721±529
EP-1(0.03mM)	16215±1921.2	1082.2±108.63	15129±1510.2	2013±449*
EP-1(0.1mM)	18250±1851.8*	1278.6±180.59	14065±1607.9	3465±868*
EP-1(0.3mM)	19100±2123.3*	1342.5±200.39*	16103±1004.3	4112±638**

*P<0.05(VS CMPS)

**P<0.01(VS CMPS).

The ORAC value, the DPPH and superoxide anion radical scavenging activity of EP-1 in the same dose, expressed as trolox equivalents, were higher than CMPS, and at the same time EP-1 shows dose-dependent character respectively. However, in the same dose the CMPS exhibited markedly higher activity for OH radical scavenging activity.

### Protective effect of EP-1 against H_2_O_2_ induced GES-1 cell death

H_2_O_2_ toxicity was first studied in GES-1 cells via MTT assay. Here, GES-1 cells incubated with various concentrations of H_2_O_2_ for 12 h exhibited decreased viability in a dose-dependent manner, with 53.2% viability at 100 mM H_2_O_2._ Thus, all subsequent experiments were carried out using 100 M H_2_O_2._

**Fig 1:**
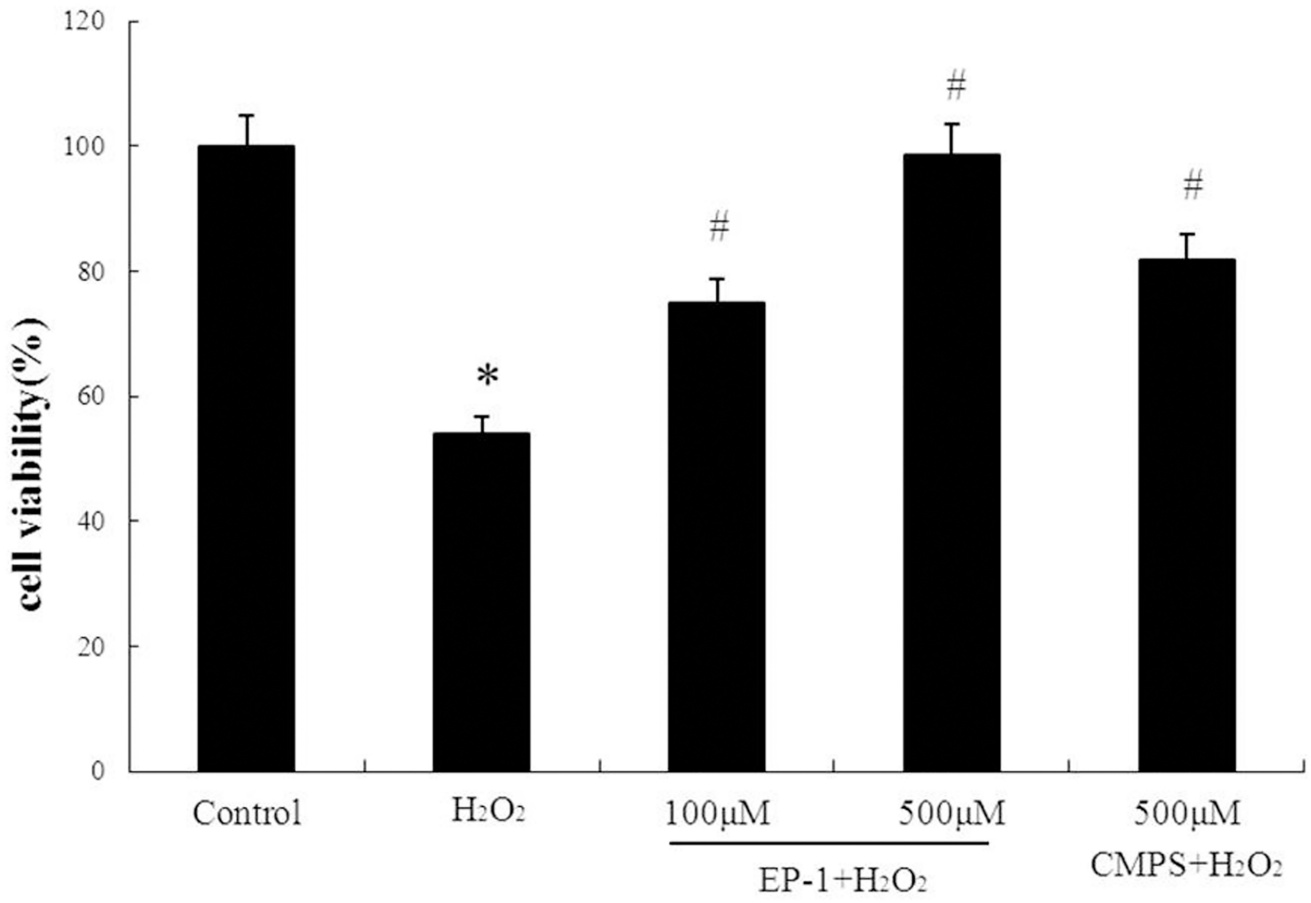
Protection of GES-1 cell by HE polysaccharides against H_2_O_2_. Values are expressed as mean ± SD of three independent experiments, *P<0.05, indicates a significant difference from the normal control group. #P <0.05, indicates a significant difference from the H_2_O_2_ group.

To study the activity of EP-1 to protect GES-1 cells against H_2_O_2_ abuse, cells were treated with various concentrations of EP-1 for 6 h prior to H_2_O_2_ treatment and then cell viability was assessed via MTT assay. It was revealed EP-1 significantly prevented H_2_O_2_-induced decreases in viability in a dose-dependent manner (Fig 1B) with decreases of 74.9% and 98.6% observed for 100 and 500 μg/mL EP-1 concentrations, respectively. CMPS also prevented H_2_O_2_-induced cell death, but its activity was significantly weaker than that of EP-1 when both were assayed at 500 μg/mL.

### Prevention of H_2_0_2_ mediated apoptosis in GES 1 cells by EP-1

**Fig 2:**
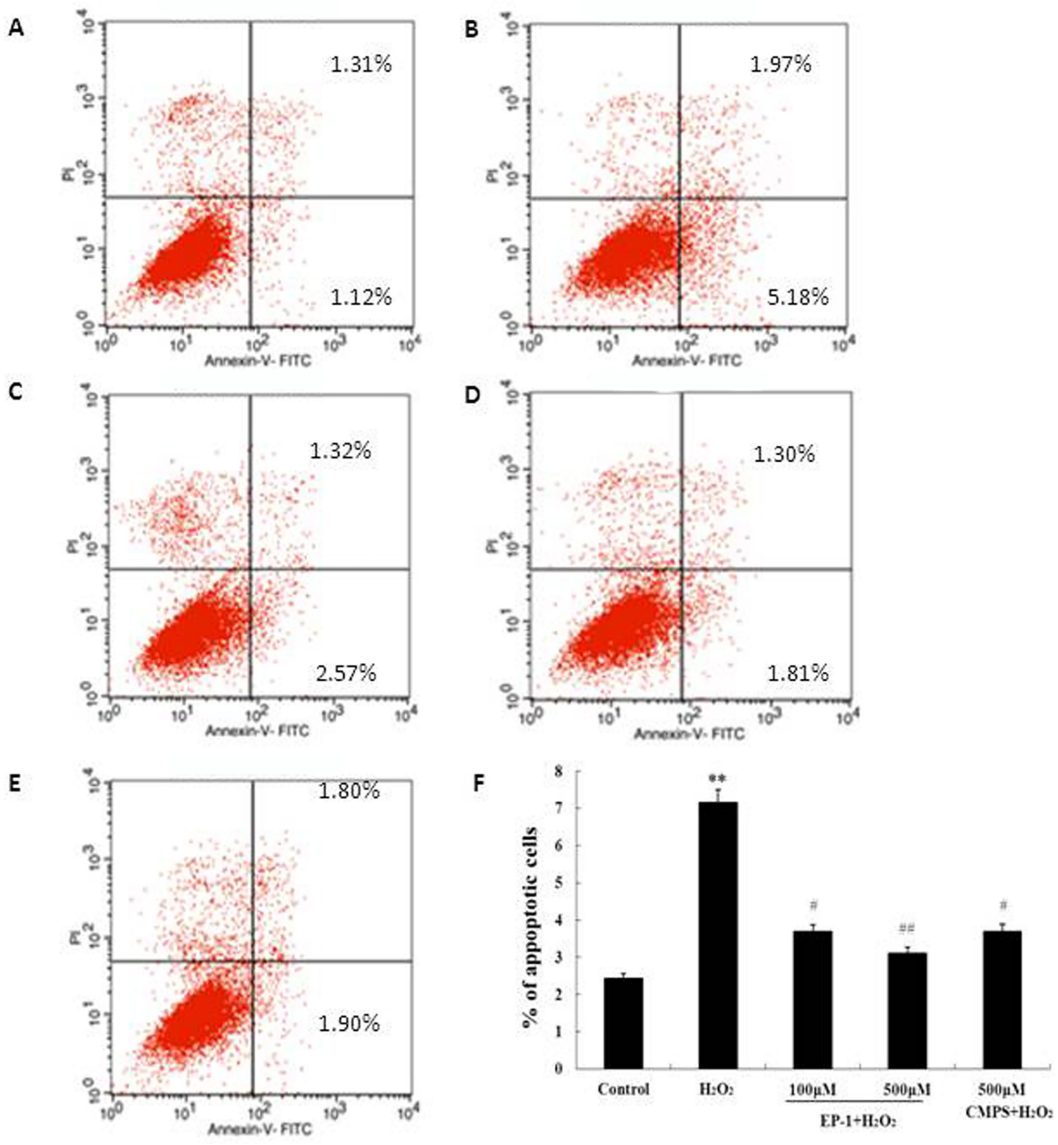
**Prevention of H2O2 induced apoptosis in GES-1 cell by EP-1.** (A)The normal control group; (B) H2O2 group; (C) EP-1 (100μM) + H2O2;(D) EP-1 (500μM) + H2O2; (E) Positive Control (500μM) + H2O2; (F) Typical histogram of apottotic ratio determined using Annexin-ⅴ/PI Apoptosis Detection Kit. Values are expressed as mean ± SD of three independent experiments, **P<0.01 versus normal control group; #P<0.05, ##P<0.01 versus H2O2 group.

To further understand how EP-1 prevents H_2_O_2_-induced cell death, annexin V-sensitive cells, serving as a pro-apoptotic cell model, were analyzed by flow cytometry (Fig 1). Cells treated with EP-1 (100 or 500 μg/mL) for 6 h, followed by H_2_O_2_ (100 μM) treatment for 12 h were labeled using an Annexin V-FITC Apoptosis Detection Kit (Beyotime Inst. Biotech) and subjected to FACS analysis as described above. The apoptotic cell fraction increased to 9.5% after H_2_O_2_ treatment, compared to 1.3% in the untreated control, but the increase was significantly inhibited in cells pretreated with EP-1 and the preventive effect was dose dependent. CMPS also reduced the apoptotic cell fraction, but the reduction was less than that observed for EP-1 (Fig 2).

### Effect of EP-1 on MDA level and GSH in GES-1 cells exposed to H_2_O_2_

**Fig 3:**
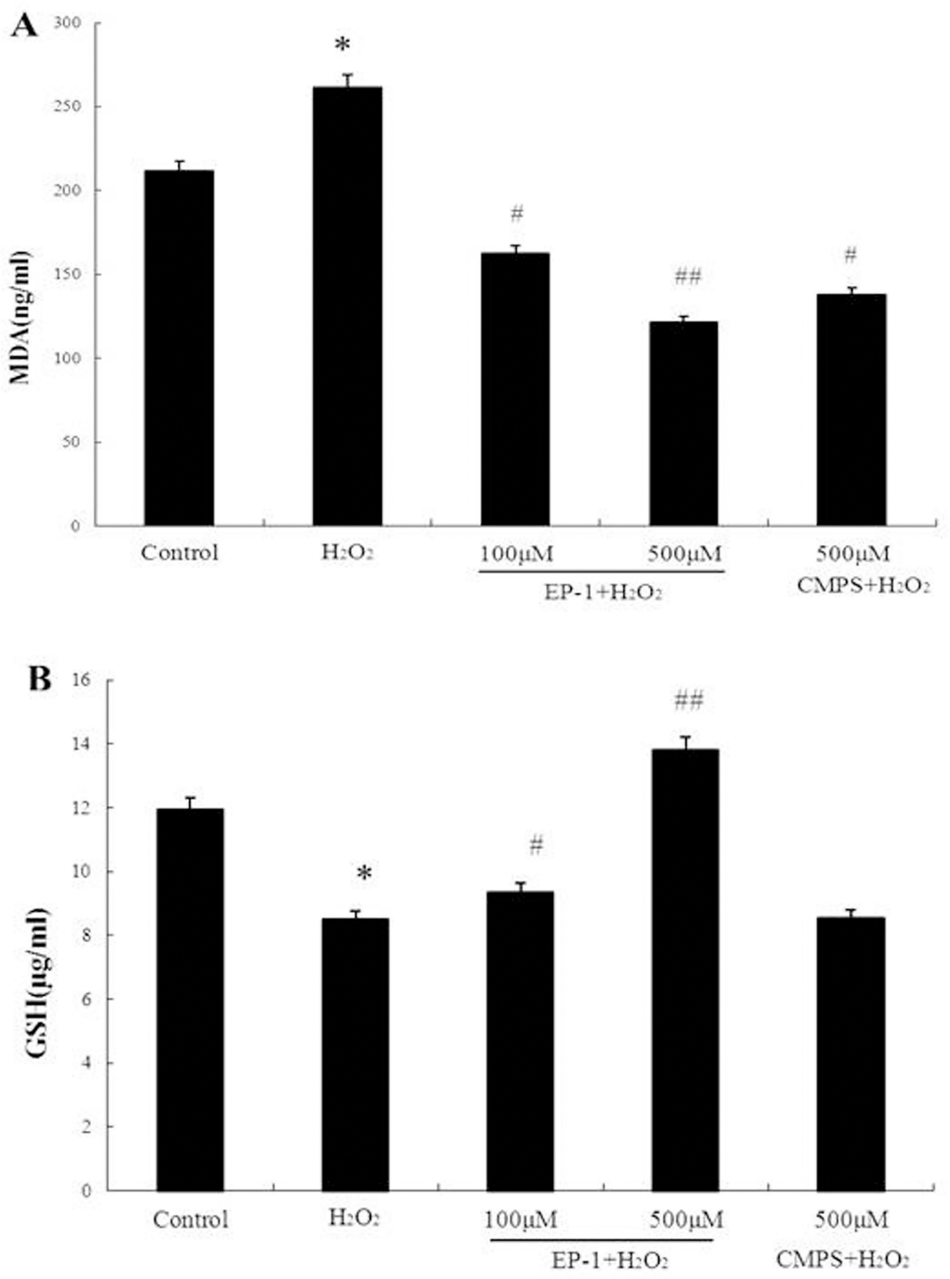
The MDA and GSH level of EP-1 on the apoptosis of GES-1 exposed to H_2_O_2_. (A) Following pretreatment with different concentrations of EP-1 for 6 h, the GES-1 cells were treated with H_2_O_2_ (100μM) for an additional 12 h, the level of cellular GSH was determined. **P<0.01 indicates a highly significant difference from the control group. #P <0.05 indicates a significant difference from the H_2_O_2_-treated group. (B) Following pretreatment with different concentrations of EP-1 for 6 h, the GES-1 cells were treated with H_2_O_2_ (100μM) for an additional 12 h, the level of MDA in the medium was determined. *P<0.05 indicates a significant difference from the control group. #P <0.05 indicates a significant difference from the H_2_O_2_-treated group.

MDA is one of the end-products of lipid peroxidation, the main event in free radical-induced cell injury, and is widely used as a bio-marker of oxidative stress [15]. MDA formation increased after H_2_O_2_ treatment (16.2 nmol/mg protein vs. 12.7 nmol/mg protein in the control) as reported elsewhere, but was significantly inhibited in cells pretreated with EP-1 (14.4 and 15.7 nmol/mg at 100 and 500 μg/mL EP-1, respectively). When the inhibition of MDA production by CMPS was compared with EP-1 at 0.5 mg/mL, CMPS activity was slightly weaker (Fig 3A).

The evidence for antioxidant activity involvement in cell protection was strengthened when cellular GSH levels were assayed. GSH is a physiological antioxidant playing a major role in defense against oxidative stress [16]. In GES-1 cells treated with 100 μM H_2_O_2_ for 12 h, GSH levels significantly decreased from 185.4 nmol/mg protein in the control to 61.8 nmol/mg protein (Fig 3B). However, in GES-1 cells pretreated with EP-1, GSH levels did not decrease as much and the effect was dose-dependent (93.6 ± 4.21 and 187.1 ± 3.95 nmol/mg at EP-1 concentrations of 0.1 and 0.5 mg/mL, respectively) whereby the GSH level remained at the control level at the higher EP-1 concentration. It is noteworthy that the effect of CMPS on GSH level was considerably less apparent than that of EP-1, in contrast to the results obtained for apoptotic protection and MDA inhibition.

### Effect of EP-1 on cell signals related to apoptosis determined by Western blot

**Fig 4:**
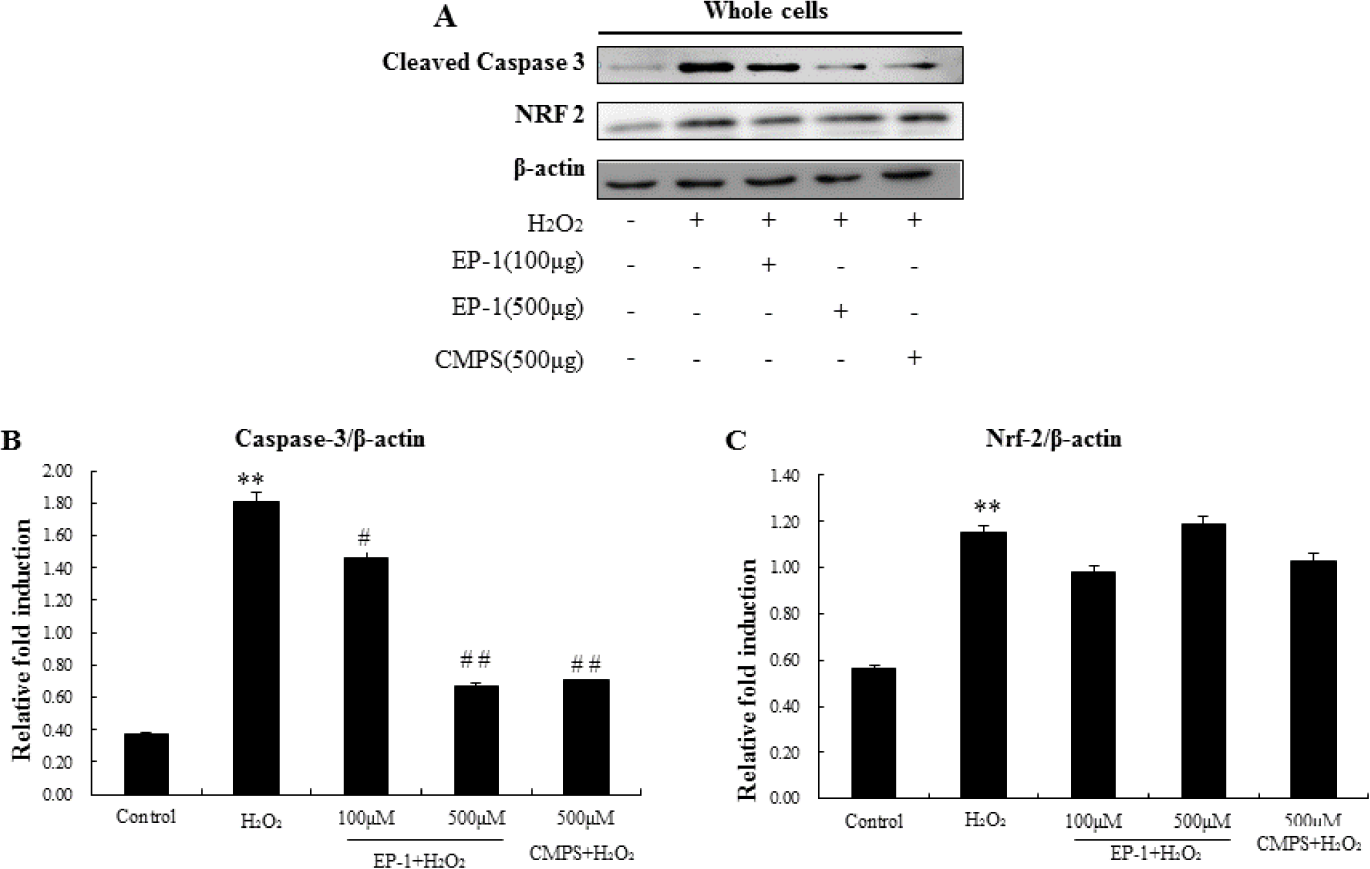
Western blotting analysis of apoptosis related cellular signals. (A) Representative western blotting of Nrf-2 and Caspase-3 with β-actin as the internal control.(B) and (C) Expression ration of Caspase 3 and NRF2. Values represent mean ± SD of three independent experiments, *P<0.05, **P<0.01 versus normal control group; #P<0.05, ##P<0.01 versus H_2_O_2_ group.

The pathway of H_2_O_2_-induced apoptosis may be caused by Caspase and Nrf2 families. For further confirmation that EP-1 prevented H_2_O_2_-induced apoptosis signals in GES-1 cells, three possible signalling pathways were investigated. The impact of EP-1 on the expression of the three cellular apoptotic signals was studied using western blot analysis (Fig 4A).

The results showed that caspase 3 apoptosis marker proteins were overexpressed in H_2_O_2_-treated cells, levels of both were inhibited by EP-1 and CMPS, The EP-1 also showed a dose-dependent manner (Fig 4B). The effects of EP-1 and CMPS on upstream signals Nrf2 were further examined. Nrf2 expression was significantly reduced in H_2_O_2_-treated cells, but was not recovered by addition of EP-1 and CMPS (Fig 4C). The results showed that anti-apoptosis induced by oxygen stress of the polysaccharide might more related by caspase 3.

The caspase 3 activation pathway that begins with mitochondrial damage followed by cytochrome c.

Mitochondria are both a major endogenous source and target of ROS, and oxidative stress has been shown to induce apoptotic cell death by targeting the mitochondria directly. Mitochondrial-dependent apoptosis has been shown to require release of cytochrome c from mitochondria and subsequent activation of a specific class of cytoplasmic proteases known as caspases 3. Bcl-2, an anti-apoptotic protein localized to mitochondria, has been shown to inhibit cytochrome c release and protect against oxidative stress-induced apoptosis [17].

**Fig 5:**
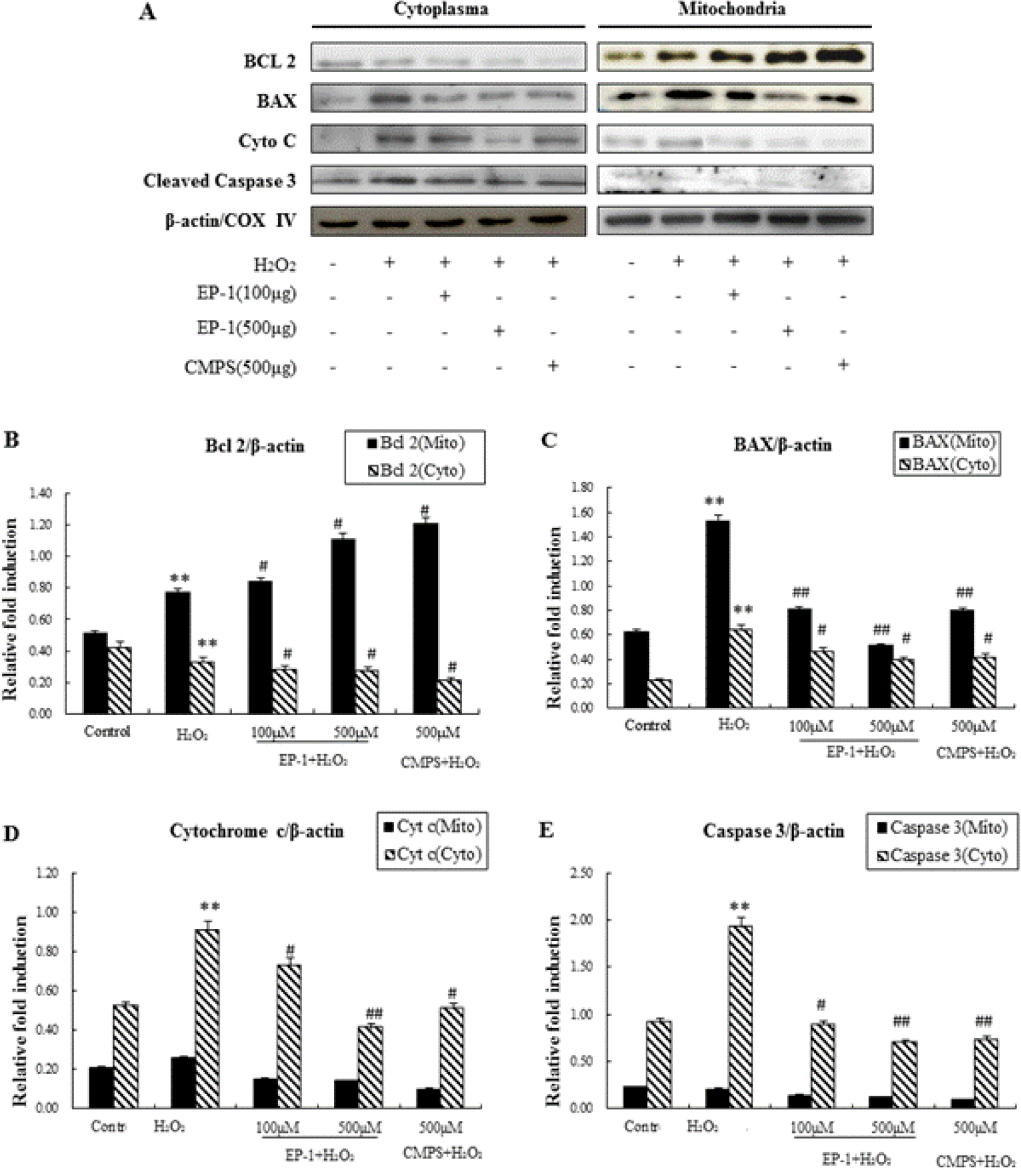
Western blotting analysis of mitochondria-related apoptosis. (A) Representative western blotting of Nrf-2, Caspase-3, Bcl-2, Bax and Cytochrome c with β-actin and COX IV as the internal control. (B)-(E) Expression ration of Bcl 2, BAX, Cytochrome c and Caspase 3. Values represent mean ± SD of three independent experiments, *P<0.05, **P<0.01 versus normal control group; #P<0.05, ##P<0.01 versus H_2_O_2_ group.

To further characterize the mechanisms for the mitochondria-related apoptosis induced in GES-1 cells, the cytoplasm and mitochondria were separated. The protein expression for Caspase-3, Cyto C, Bax, and Bcl-2 (in the cytoplasm and in the mitochondria) was measured by Western Blot analysis (Fig 5).

The results showed that cytochrome c and Caspase 3 were not released from mitochondria to cytosol (Fig 5D and 5E). In the Western blot analysis shows although in the both cytochrome c in the in the cytoplasm and Bax in the mitochondria, also overexpressed in H_2_O_2_-treated cells, were also suppressed by EP-1 (Fig 5C). However, in the mitochondria Bcl-2, an apoptosis suppressor molecule, was also significantly decreased by EP-1 in a dose-dependent manner, even though EP-1 decreases apoptosis. Moreover, Bcl-2 showed higher expression in H_2_O_2_-treated cells without EP-1 (Fig 5B). These results suggest EP-1 can inhibit H_2_O_2_-induced apoptosis in GES-1 cells by mitochondria-dependent apoptotic pathways.

## Discussion

Maintaining redox conditions in cells is important for cellular physiology. Oxidative stress is a condition whereby the redox balance shifts to oxidized conditions due to the excess production of reactive oxygen species (ROS) by accidental or physiological processes, resulting in damage to cells and tissues[18,19].Among the ROS, H_2_O_2_ is important because it is rather stable over a large tissue area and it produces highly reactive species, such as hydroxyl radicals, via the Fenton reaction[20].Moreover, H_2_O_2_ also oxidizes biologically important molecules such as proteins[21]. Therefore, H_2_O_2_-induced oxidative injury provides a model for studying the protective effects of antioxidants.

HE has exhibited anti-gastric ulcer and anti-cancer effects, but the active principle has not yet been elucidated. In our previous study, we established the antioxidant properties of both a crude polysaccharide-rich extract of HE mycelium and a purified fraction of this extract using a model of H_2_O_2_-induced injury of human gastric mucosal epithelial cells (GES-1). Moreover, we isolated a polysaccharide molecule (EP-1) with a molecular weight of 3 KDa from the mycelium of HE and showed that EP-1 prevented anti-gastric ulcer activity in both EtOH-induced gastric ulcer animal and cell models[3,4]. Since oxidative stress and inflammation play critical roles in pathogenesis of gastric ulcers caused by various stimuli, including Helicobacter pylori [22], studies of the antioxidant properties of EP-1 should be of value. Moreover, the antioxidant potential of polysaccharides has not been as extensively studied as has been done for low molecular weight ingredients, such as polyphenols [23].

Because the results of our previous study indicated that CMPS possessed antioxidant properties and was effective against H_2_O_2_-induced GES-1 cell injury, we investigated antioxidant properties further in this work. The results showed that an extract of CMPS, EP-1, prevented elevation of cellular malondialdehyde (MDA) levels by enhancing the activity of glutathione peroxidase (GPx), thereby increasing cellular GSH levels. Moreover, EP-1 successfully prevented H_2_O_2_-induced apoptotic cell death in GES-1 cells through modulation of cellular apoptosis signal molecules. Specifically, EP-1 suppressed overexpression of Bax, caspase 3 and cytochrome c, all apoptosis-promoting signals, while preventing downregulation of apoptosis-blocking molecules such as Bcl-2. Therefore, these data strongly and collectively suggest that EP-1antioxidant activity prevents apoptotic cell death of GES-1 cells by modulating cellular redox conditions.

It is interesting to note that CMPS, which has antioxidant potential comparable to that of EP-1, also showed similar antioxidant protection and apoptosis inhibition. Thus, polysaccharides may generally play significant roles in preventing ulcers through antioxidant effects. However, the protective potential of CMPS against H_2_O_2_ toxicity in GES-1 cells was weaker than for EP-1 at the same treatment dose (500 μg/mL). This differential effect between EP-1 and CMPS was most pronounced for GSH modulating activity. Although EP-1 prevented H_2_O_2_-dependent decreases in cellular GSH levels and even increased the level above that of the control, CMPS did not show any significant effect on GSH level, although CMPS prevented MDA level decreases comparable to decreases observed using EP-1. Moreover, several differences in cellular signal-modulating activities between EP-1 and CMPS were also observed, such that EP-1 stimulated Nrf2 expression but CMPS did not. Moreover, EP-1 downregulated cytochrome c levels significantly more strongly than did CMPS, indicating their modes of antioxidant protection from H_2_O_2_ abuse differ in GES cells. Since the anti-gastric ulcer activity of EP-1 is significantly higher than that of CM*PS*, observations reported here strongly indicate that the structure of EP-1 may play a critical role in its activity that is supplemental to its known antioxidant activity. Therefore, further studies of EP-1 are planned to unravel the full potential of this promising extract for treatment of stomach disorders.

## Conclusions

In the present study, we reported antioxidant activity of the polysaccharide EP-1 isolated from HE mycelia culture *in vitro*, which structural feature and its anti-CAG have been published previously. These results suggested that EP-1 dose dependently prevented oxidative stress induced by H_2_O_2_ through mitochondrial dependent apoptotic pathway in GES-1 cells.
